# Reply to Wu, S.-N.; Tsai, C.-H. Comment on “Can Thrombosed Abdominal Aortic Dissecting Aneurysm Cause Mesenteric Artery Thrombosis and Ischemic Colitis?—A Case Report and a Review of Literature. *J. Clin. Med.* 2025, *14*, 3092”

**DOI:** 10.3390/jcm14134698

**Published:** 2025-07-03

**Authors:** Laurențiu Augustus Barbu, Nicolae-Dragoș Mărgăritescu, Liliana Cercelaru, Daniel-Cosmin Caragea, Ionică-Daniel Vîlcea, Valeriu Șurlin, Stelian-Ștefaniță Mogoantă, Gabriel Florin Răzvan Mogoș, Liviu Vasile, Tiberiu Ștefăniță Țenea Cojan

**Affiliations:** 1 Department of Surgery, Railway Clinical Hospital Craiova, University of Medicine and Pharmacy of Craiova, 2 Petru Rareş Street, 200349 Craiova, Romania; laurentiu.barbu@umfcv.ro (L.A.B.); gabriel.mogos@umfcv.ro (G.F.R.M.); tiberiu.tenea@umfcv.ro (T.Ș.Ț.C.); 2 Department of Surgery, Emergency County Hospital, University of Medicine and Pharmacy of Craiova, 2 Petru Rareş Street, 200349 Craiova, Romania; ionica.vilcea@umfcv.ro (I.-D.V.); vsurlin@gmail.com (V.Ș.); ssmogo@yahoo.com (S.-Ș.M.); vliviu777@yahoo.com (L.V.); 3 Department of Pathology, University of Medicine and Pharmacy of Craiova, 200349 Craiova, Romania; liliana.cercelaru@umfcv.ro; 4 Departament of Internal Medicine, University of Medicine and Pharmacy of Craiova, 200349 Craiova, Romania; daniel.caragea@umfcv.ro

We sincerely thank the authors for their interest in our article titled “Can Thrombosed Abdominal Aortic Dissecting Aneurysm Cause Mesenteric Artery Thrombosis and Ischemic Colitis?—A Case Report and a Review of Literature”, and we appreciate their thoughtful engagement with our work [1]. We welcome the opportunity to clarify the points they have raised and to contribute further to the academic discussion on this complex and rare vascular pathology.

**1. Regarding the omission of detailed peripheral vascular examination findings:**

We acknowledge the reviewer’s emphasis on the importance of peripheral pulse assessment. Although such an examination was performed at presentation and yielded no abnormal findings, these were not detailed in the report due to their lack of diagnostic significance in the acute setting. We agree that including these results would have strengthened the clinical narrative, and we will aim to address this more explicitly in future reports.

**2. On the potential risk of inappropriate resuscitation in undiagnosed vascular disease:**

We appreciate the comment highlighting the relevance of peripheral vascular assessment in minimizing risks during cardiopulmonary resuscitation. While our patient was not diagnosed with peripheral arterial disease, we agree that early identification of such conditions may inform clinical decision-making in emergency situations [2].

**3. Concerning the etiology of the abdominal aortic aneurysm:**

Our reference to the unclear etiology was specific to this individual case where no single definitive cause could be determined. The patient had no documented history of chronic hypertension, connective tissue disorders, or untreated infections such as syphilis. While we acknowledge the relevance of these conditions as potential contributors, none were evident in the patient’s history or clinical profile.

**4. Regarding the possibility of Marfan syndrome:**

There were no phenotypic or clinical features suggestive of Marfan syndrome in this case. The patient lacked the characteristic body habitus, ocular findings, or family history typically associated with the syndrome. We concur that Marfan syndrome is usually diagnosed earlier in life and is unlikely to go unnoticed in a patient of advanced age with long-term cardiological follow-up.

**5. On the diagnostic algorithm and patient comorbidities:**

We agree that a more detailed presentation of the diagnostic process could have enhanced the case description. The patient had a long-standing history of cardiovascular disease and had undergone extensive diagnostic evaluations prior to this acute event. While rare genetic or non-genetic factors may contribute to vascular pathology in elderly patients, no such evidence was present in this particular case.

We will ensure that future submissions include a more detailed and structured presentation of the patient’s history and clinical examination along with clear diagnostic flowcharts to improve clarity and clinical coherence.

We once again thank the authors for their engagement with our work. Exchanges such as this are vital to the continued advancement of clinical knowledge and practice. We hope our responses provide clarity and welcome further discussion if needed.
